# Association of Sex Hormones and Androgens with Disease Activity in Premenopausal Females with Rheumatoid Arthritis

**DOI:** 10.31138/mjr.34.2.152

**Published:** 2023-06-30

**Authors:** Pulin Kumar Gupta, Ankita Sheoran, Pankaj Gupta, Subodh Kumar Mahto, Princi Jain, A.K. Varshney, Lokesh Kumar Sharma

**Affiliations:** ABVIMS & Dr. RML Hospital, New Delhi, India

**Keywords:** sex hormones, androgens, premenopausal women, rheumatoid arthritis, disease activity

## Abstract

**Background:**

Gonadal sex hormone dysfunction is frequently reported in patients with Rheumatoid arthritis (RA). The relationship of these hormones with disease activity is still not clear and whether the hormone imbalance leads to increased severity of RA is not well studied in this part of the world. The present study aimed to elucidate this fact.

**Methods:**

It was a cross-sectional observational study performed in 80 premenopausal females with definite RA at a tertiary care hospital in New Delhi, India over one year. Patients were subjected to investigations as per the protocol and a fasting venous blood sample for hormone levels was collected in the follicular phase of their menstrual cycle.

**Results:**

A statistically significant correlation by linear logistic regression analysis was found between disease activity (as measured by DAS28) and serum progesterone, FSH, and prolactin, while serum testosterone and DHEAS showed an inverse relationship with disease activity. Low s. prolactin, and s. FSH as well as high s. testosterone and s. DHEAS were found to be associated with target clinical goals in RA (ie, remission and low disease activity). On multivariate logistic regression analysis, serum prolactin showed a direct association. (p=0.016, OR= 1.009. C.I.= 1.0021.017) and serum testosterone were found to have an inverse relationship (p=0.002, OR= 0.017, C.I.=0.001–0.237) with disease activity in this group of individuals.

**Conclusion:**

Serum levels of sex hormones may be helpful in predicting disease activity among patients with RA, and in future, may be used to guide treatment of severe refractory disease, unresponsive to conventional treatment with DMARDs, especially in resource-poor settings.

## KEY MESSAGES:

- Androgens in males are postulated to be protective against the development of various immuno-inflammatory diseases like RA. However, the relationship of androgens with disease activity is still unclear. We could not find any study collectively correlating all these hormones with disease activity; hence the study was carried out.
- The levels of LH, FSH and prolactin were significantly higher in patients with moderate/high disease activity compared to those on remission/low disease activity. Also, testosterone and DHEAS were significantly higher in patients with remission/low disease activity versus those with moderate/high disease activity. Surprisingly, contrary to the available literature, no correlation was found between the levels of serum oestrogen and progesterone with DAS-28 in our cases.
- Serum levels of sex hormones may be helpful in predicting disease activity among patients with RA, and in future, may be used to guide treatment of severe refractory disease unresponsive to conventional treatment with DMARDs when cost limits biological therapy.

## INTRODUCTION

Rheumatoid arthritis (RA) is a chronic inflammatory disease of autoimmune aetiology marked by symmetric, peripheral polyarthritis. The prevalence of RA in females has increased from 0.8% (7.7 per 1000) in 1995 to 1% (9.8 per 1000) in 2005 but the prevalence has remained constant among males in both 1995 and 2005 at 0.4%.^1^ The incidence rate increased by 2.5% each year from 1995 to 2005 among females but decreased by 0.5% per year among males.^1^ Men, as well as premenopausal women suffering from RA have been found to have lower levels of mean serum testosterone and DHEAS than healthy controls. Whether the androgen deficiency is the result of RA or a contributing factor to the disease pathology is still unknown. Similarly, the correlation of higher oestrogen, progesterone, prolactin, LH, and FSH levels with disease activity of RA has been conflicting. The postulated reason behind the increased prevalence of RA in females is that androgens in males are protective against the development of various immuno-inflammatory diseases like RA.^2^ However, the relationship of these hormones with disease activity till now is still not clear. We could not find any study collectively correlating all these hormones with disease activity. Hence, the present study was planned and executed at a tertiary care centre in New Delhi, India.

## METHODS

It was a cross-sectional observational study done in 80 premenopausal females with definite RA as per ACREULAR 2010 criteria, recruited over one year after obtaining ethical and institutional review board permission. All cases with a history of coronary artery disease, chronic liver or kidney disease, diabetes, hypothyroidism, hyperthyroidism, hysterectomy, or oophorectomy were excluded. Similarly, patients on oral contraceptive pills, intrauterine contraceptive device (IUCD), testosterone injection, ongoing biological therapy, or those who had taken corticosteroids within the last 3 months were excluded. Patients were subjected to investigations as per a defined protocol. The hormone levels were measured in the follicular phase of the menstrual cycle using the VITROS ECI/ECIQ Immunodiagnostic systems, using Intellicheck Technology. After taking aseptic precautions, around 10ml of fasting venous blood was sampled from each subject. A competitive immunoassay technique was used to measure serum oestradiol, progesterone, and testosterone levels. An immunometric immunoassay technique was used to measure serum FSH, LH, and Prolactin. The Calbiotech Inc. (CBI) DHEA-S ELISA kit was used for the quantitative measurement of DHEA-S and was based on the principle of competitive binding between DHEA-S in the test specimen. The laboratory reference range for each of the measured hormones was as follows- Oestradiol: 97.5–592 pmol/L, Progesterone: 0.44–6.47 nmol/L, FSH= 1.3–23.4 mIU/mL, LH= 0.8–15.5 mIU/ml, Prolactin= 3–18.6 ng/mL, Testosterone=0.198–2.67 nmol/L, DHEAS=0.48–2.75 μg/mL.

Complete blood count was analysed by Autoanalyzer Medtronic CA620. Quantitative CRP was estimated by the immunoturbidimetry method. Rheumatoid factor was estimated by ELISA. Anti-CCP antibodies were measured by second-generation ELISA test using AUTOSTATTM kit.

### Statistical analysis

Categorical variables were presented in number and percentage (%) and continuous variables were presented as mean ± SD. Normality of data was tested by Kolmogorov-Smirnov test. If the normality was rejected, then a non-parametric test was used. Quantitative variables were compared using Mann-Whitney Test (as the data sets were not normally distributed) between the two groups.

Spearman rank correlation coefficient was used to assess the association of various parameters with DAS 28. A p-value of <0.05 was considered statistically significant. The data was entered in MS EXCEL spreadsheet and analysis was done using Statistical Package for Social Sciences (SPSS) version 21.0. The association of serum levels of sex hormones as independent variables with increased DAS28 as dependent variable was initially assessed by univariate regression analysis. Further, variables found to have association with DAS28 on univariate analysis were subjected to multivariate regression analysis to determine the independent association of the variable with DAS28.

Univariate linear regression analysis was done with DAS28 as a dependent variable and oestradiol, progesterone, FSH, LH, prolactin, testosterone, and DHEAS as independent variables.

On multivariate analysis, anti-CCP, FSH, oestradiol, and progesterone were found to have a statistically significant direct association with DAS28, however LH, prolactin, testosterone, and DHEA did not have association with DAS28 due to some other cofounding variables.

## RESULTS

This was a cross-sectional observational study carried out in 80 premenopausal females with RA. The mean age of the cases was 35.2 ± 5.35 years. The mean duration of the disease was 4.6 ± 0.8 years. Seropositivity and rheumatoid related deformities were found in 97.5% and 12.5% patients, respectively. The mean DAS −28 was 3.43 ± 0.7. The mean CRP was 2.26 ± 1.72 mg/dl (range 0.21 – 6.4 mg/dl). The mean ESR was 33 ± 21.06 mm/hr (range 2–78 mm/hr). The mean anti-CCP was 70.77 ± 16.05 (range 5–87). All patients were receiving similar treatment, which was methotrexate-based DMARDS with nearly 80% on triple-drug therapy constituting hydroxychloroquine, sulfasalazine, and methotrexate.

The mean value of sex hormones and androgens in 80 cases with RA has been depicted in **Table 1.** Univariate logistic regression analysis amongst all cases (**Table 2**) revealed a statistically significant direct association of serum FSH (p=0.013, OR=1.021, C.I.=1.004–1.037), serum progesterone (p=0.034, OR=1.008. CI=1.001–1.016) and serum prolactin (p=0.013, OR=1.007, CI=1.003–1.012) with disease activity as measured by DAS-28. On the other hand, serum testosterone (p=0.002, OR=0.03, CI=0.0030.269) and serum DHEAS (p=0.005, OR=0.236, CI=0.119–0.688) were found to have a significant inverse relationship with disease activity.

**Table 1.** Mean value of sex hormones in cases.

**Parameters**	**Mean ± Sd**	**Median**	**Min-Max**	**Interquartile Range**
LH (mIU/ml)	20.35 ± 24.76	8.38	0.22–96.9	4.090 – 29.150
FSH (mIU/ml)	24.49 ± 32.31	7.175	2.03–140.2	4.488 – 36.075
17 beta estradiol (pmol/L)	282.4 ± 245.46	200.85	1.37–906.4	80.500 – 422.180
Progesterone (nmol/L)	37.92 ± 82.54	31.205	0.25–350	1.753 – 23.25
Testosterone (nmol/L)	0.61 ± 0.84	0.42	0.01–6.44	0.193 – 0.635
DHEAS (μg/ml)	0.74 ± 0.63	0.59	0.01–2.64	0.183 – 1.205
Prolactin (ng/ml)	62.64±140.43	13.95	5.1–810.7	9.18 – 44.5

**Table 2.** Univariate logistic regression analysis of association between sex hormones and DAS-28.

**Parameters**	**p value**	**ODD’s ratio (OR)**	**Coefficient Interval (C.I)**
**LH**	0.065	1.018	0.99 – 1.037
**FSH**	**0.013**	1.021	1.004 –1.037
**17 beta estradiol**	0.194	1.001	0.99 – 1.003
**Progesterone**	**0.034**	1.008	1.001 –1.016
**Testosterone**	**0.002**	0.03	0.003 – 0.269
**DHEAS**	**0.005**	0.236	0.119 – 0.688
**Prolactin**	**0.013**	1.007	1.003 – 1.012

However, on multivariate logistic regression analysis (**Table 3**), only serum prolactin (p=0.016, adjusted OR=1.009, CI=1.002–1.017) was found to have a direct correlation with DAS-28 and serum testosterone (p=0.002, adjusted OR=0.017, CI=0.001–0.237) was found to have a significant inverse relationship with DAS-28.

**Table 3.** Multivariate logistic regression analysis of sex hormones with DAS-28.

**Parameter**	**P value**	**Adjusted odds ratio**	**C.I.**
**PROLACTIN**	**0.016**	1.009	1.002 – 1.017
**TESTOSTERONE**	**0.002**	0.017	.001 – 0.237

The proportion of cases in remission, low disease activity (LDA), moderate disease activity (MDA), and high disease activity (HDA) were 45%, 15%, 21%, and 19% respectively. The mean value of sex hormones in different disease categories is illustrated in (**Table 4**). The differences in the values of serum prolactin, testosterone, and DHEAS between different disease categories were statistically significant. Those with higher disease activity had increased levels of serum prolactin, whereas higher levels of serum testosterone and DHEAS were found to predict less severe disease. A direct correlation was seen to exist between moderate to severe disease with high serum prolactin (p= 0.022, r=0.257) and low serum testosterone (p= 0.001, r= 0.344), and DHEAS (p= 0.001, r=0.275).

**Table 4.** Mean value of sex hormones amongst groups graded as per disease activity by DAS-28.

**Parameters**	**Remission (DAS28<2.6)**	**Low Disease Activity (2.6-<3.2)**	**Moderate Disease Activity (3.2<5.1)**	**High Disease Activity (≥5.1)**	**P value**
**LH**	16.69	11.46	25.26	29.43	0.210
**FSH**	16.39	14.67	37.34	35.61	0.152
**17 beta estradiol**	225.14	344.28	322.87	333.83	0.212
**Progesterone**	19.83	10.57	48.47	88.14	0.646
**Testosterone**	0.74	1.20	0.32	0.27	**0.001**
**DHEAS**	0.82	1.29	0.55	0.43	**0.003**
**Prolactin**	29.09	47.46	94.14	126.32	**0.035**

In clinical practice, while managing RA, the physician’s target of treatment is achieving either remission or at least low disease activity. Hence to compare our results which may be applicable in clinical context and day to day practical management of RA we made two subgroups, ie, cases with remission or LDA (**Group-A**, **n=48**) and cases with MDA or HDA (**Group-B, n=32**), and analysed the difference between levels of sex hormones in these two subgroups (**Table–5**).

**Table 5.** Association of sex hormones with different group of disease activities (Remission + LDA [group-A] vs MDA+HDA [group-B]) divided as per clinical goals.

**Parameter**	**Group A (n= 48)**	**Group B (n= 32)**	**P value**
**LH**	15.84 ± 21.97	26.45 ± 27.25	**0.041**
**FSH**	16.25 ± 23.02	35.64 ± 39.41	**0.025**
**17 beta estradiol**	251.66 ± 226.52	323.98 ± 266.77	0.157
**Progesterone**	18.20 ± 50.04	64.59 ± 107.80	0.221
**Testosterone**	0.84 ± 1.03	0.3 ± 0.19	**<0.001**
**DHEAS**	0.92 ± 0.66	0.5 ± 0.5	**0.001**
**Prolactin**	33.5 ± 70.79	105.86 ± 143.76	**0.022**

**Graphs 1–7.**
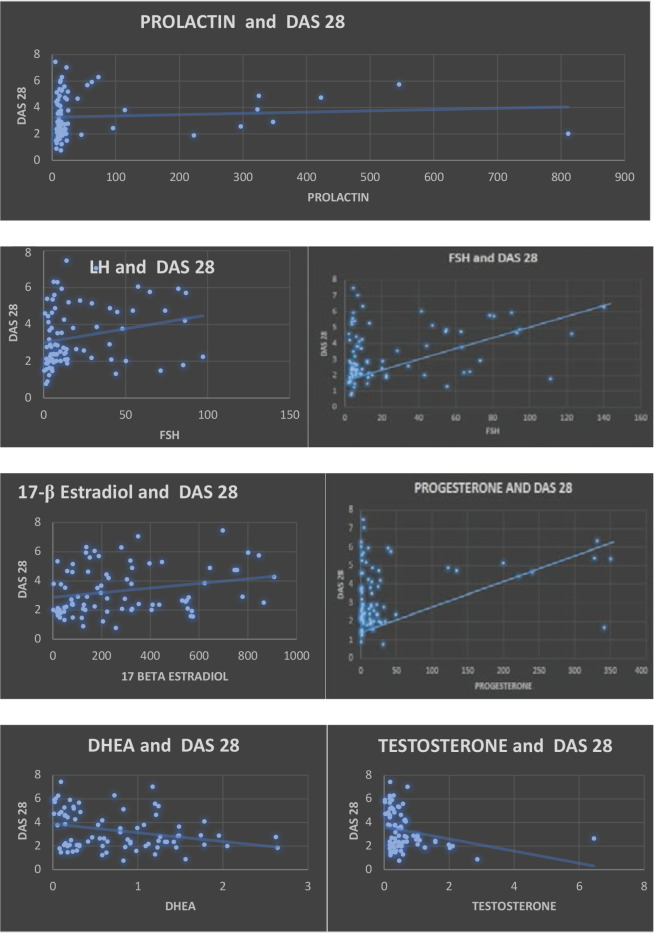
Various hormone levels and their relation with DAS28.

It was observed that higher levels of serum LH, FSH, and Prolactin were found to be directly associated with disease activity in group B, and thus, associated with moderate to high disease activity. On the other hand, higher levels of serum testosterone and DHEAS were observed to be significantly higher in patients with remission/low disease activity versus those with moderate/high disease activity **(graphs 1–7**), implying that the male sex hormones/androgens either are protective against higher grades of disease activity in RA or their insufficiency predisposes to severe disease activity. Similarly, increased levels of serum prolactin, FSH, and LH may warrant additional therapy for the disease as they correlate with severe disease activity in this group of individuals. Surprisingly, contrary to the available literature, no correlation was found between the levels of serum oestrogen and progesterone with DAS-28 in our cases. Although numerically oestrogen and progesterone levels were found to be higher in cases of group-B as compared to group-A, the difference could not achieve statistical significance.

## DISCUSSION

Hypothalamic – pituitary–adrenocortical (HPA) axis and sex hormone dysfunction has been seen to contribute to the development as well as the severity of rheumatoid arthritis. Stress and inflammation can increase HPA axis activity through the central and peripheral actions of circulating cytokines.^3^ Oestrogens have been claimed to be enhancers and androgens and progesterone as natural suppressors of immunity. Low gonadal and adrenal androgens (testosterone and dehydroepiandrosterone sulphate (DHEAS)) along with reduced androgen/oestrogen ratio has been detected in serum and synovial fluid in patients with moderate to severe RA, suggesting a definite role of these hormones. Local effects of sex hormones on cell growth and apoptosis along with their effect on the genetic promotion of Th1/Th2 cytokines have been well observed.^4^

The influence of sex hormones especially oestrogen, progesterone, and androgens in rheumatoid arthritis was stated well by M. Culoto et al. in his study where serum concentrations of DHEAS, testosterone, and progesterone were found to be reduced in patients with RA in both sexes, while oestrogen was found to be higher, suggesting its role as an immune enhancer.^5^ The postulated mechanism was correlated to the increased levels of inflammatory cytokines (ie, TNF-α, IL-6, IL-1), in RA synovitis which was thought to markedly stimulate the aromatase activity in peripheral tissues.^5^ Our study similarly emphasizes the same fact that higher levels of serum testosterone and DHEAS may predict low disease activity and vice versa and hence may be protective against severe disease. This effect may be explained by the fact that 60% of our cases were in remission or LDA, and hence with probable lower levels of cytokines like IL-1, IL-6, and TNF-α, causing minimal peripheral conversion of androgen to oestrogen, thus higher androgen levels.

Blockade of the enzyme P450c17, induced by inflammatory cytokines such as IL1β and TNF at the level of adrenals and gonads has been seen to cause reduction of DHEAS. Increased DHEAS levels following treatment with TNF antagonists suggest an improved adrenal function in patients with RA. Testosterone supplementation treatment has been successful in ameliorating symptoms in men with RA.^6^ However, we excluded all cases on biological therapy to nullify the effect of TNF-α inhibitors on aromatase activity. Hence, with our results, we can safely predict the association of higher levels of androgen directly with the satisfactory achievement of target clinical goals ie, remission and LDA even in cases on conventional DMARDs.

Oestrogen receptors are omnipresent amongst cells of the immune system, such as CD4+ and CD8+ T cells, B cells, NK cells, and macrophages, inducing intracellular signals and resulting in proliferation and differentiation. However, contrary to that, oestrogens may even reduce the T-cell population (at the level of the thymus), inhibit B-cell development (at the level of the bone marrow), and induce monocyte apoptosis suggesting dual (pro as well as anti-inflammatory) actions. As against the previously published literature where oestrogen was found to be pro-inflammatory (associated with high disease activity)^7^ and progesterone was found to be anti-inflammatory (associated inversely with high disease activity), we could not achieve any correlation of both of these hormones with disease activity on multivariate logistic analysis as well as while comparing between group A and group B. This lack of association can only be explained by the fact that the majority of our cases were adequately controlled on DMARDs, and 60% of cases were in remission or LDA. Also, the mean DAS-28, ESR, and CRP were in lower range hence cytokines (TNF-α, IL-1, IL-6) that stimulate aromatase activity (which converts androgen to oestrogens) must be low in majority of cases, resulting in lower oestrogen levels.

Progesterone has been seen to stimulate Th2 responses and blocking the differentiation of Th1 and Th17 cells.^8^ Rapid progesterone withdrawal causes upregulation of IL-1b, and TNF-α expression increasing the disease severity. However, in our study, no correlation could be established between serum progesterone levels and disease activity. The reason may be that the present study is probably amongst the fewer research where all hormones are studied collectively and correlated simultaneously (rather than female and male gonadal hormones studied separately as has been done in most studies) and serum prolactin and testosterone eclipsed the effect of progesterone. However, contrary to the common belief, the levels of serum progesterone numerically increased with higher disease activity. This is contrary to the common knowledge that low disease activity of RA in certain special situations (like pregnancy) is attributed to high progesterone levels, but we could not find such association in our cases with LDA. This can be explained by the fact that the mean progesterone level was 15.168 nmol/mL in our cases as compared to much higher concentration (up to 80nmol/mL) found in pregnancy. Thus, progesterone might require a pregnancy-like higher concentration to exert its anti-inflammatory effect.

In our study, serum prolactin was found to be directly associated with increasing grades of disease activity. Rheumatoid synovial T-cells are believed to produce prolactin in minor amounts. The addition of prolactin to synovial cells in rats suffering from RA was associated with increased production of proteolytic enzymes increasing cytokine production and thus causing cartilage destruction, suggesting the pro-inflammatory role of prolactin in RA.^9^ Bromocriptine leading to reduction of humoral and cell-mediated immunity and the benefit reversible with addition of prolactin was proven way back by Nagy et al.^10^ in 1978.

Menopause-related factors (early menopause, post-menopausal status) have been seen to be associated with the resurgence of higher disease grades of RA and higher prolactin levels have been postulated to be the aetiology. There is evidence that suggests a role of prolactin favouring a Th1 versus Th2 response. This Th1/Th2 paradigm is relevant to autoimmune disease because the Th2 phenotype favours a humoral or antibody-mediated immune response, and RA being a Th1-mediated process, suggests that promoting a Th2 response (like that seen in pregnancy) would lead to amelioration of the disease.^11^

In our study, serum LH and FSH were found to be significantly higher amongst cases in group B (ie, MDA + HDA) as compared to cases in group A (remission + LDA) indicating that excess of these hormones predisposes towards severe disease. Kass AS et al. also stated that the increases in LH and FSH correlated positively with an increase in key pro-inflammatory cytokines including TNFα, interleukin 1 beta, IL-2, IL-2R, IL-8, and many other pro-inflammatory cytokines.^12^ That is probably the reason behind the worsening of rheumatoid arthritis in the post-partum period and menopause (ie, periods of high LH and FSH).^13^

However, our study had certain limitations. We could not ascertain a causal relationship between levels of sex hormones and severity of disease due to the cross-sectional observational nature of the study. We may need a prospective interventional study to clarify the fact whether alteration of sex hormones can alter the disease severity. Secondly, we did not measure cytokine levels (especially IL-1, IL-6, TNF-α), measurement of which could have better thrown light on the lack of correlation seen especially with oestrogen and progesterone with disease activity in our study. Exclusion of patients with irregular menses, those on oral contraceptives or with history of gynaecological surgery makes it difficult to apply the observations clinically in such patients. Also, a lesser number of cases in MDA or HDA could have brought some bias in the results.

## CONCLUSION

There have been multiple studies done on individual sex hormones and their correlation with disease activity in premenopausal women with RA, however, no study has collectively studied the correlation of various sex hormones together in premenopausal women with RA and their correlation with disease activity. Our study reveals that serum prolactin is directly and androgens are inversely correlated with disease activity in premenopausal females with RA.

Univariate linear regression analysis revealed a statistically significant correlation between DAS28 and serum FSH (p=0.013), progesterone (p=0.034) and serum prolactin (p=0.013), while serum testosterone (p=0.002) and DHEAS (p=0.005) showed a significant inverse correlation with DAS28.

On multivariate analysis, serum prolactin was found to have a significant direct association and serum testosterone had a significant inverse association with DAS28. This observation may have a huge impact on the management of RA in the future. Cheaper and easily available drugs like bromocriptine, may be further investigated and tried in the management of refractory RA. Similarly, testosterone supplementation, which has no significant side effects in females (contrary to seen in males) might become a part of the treatment protocol of RA in these females with documented deficiency of this hormone.

## KEY MESSAGE

Serum levels of sex hormones may prove to be a useful tool for prediction of disease activity among patients with RA and in the future may be used to guide treatment of severe refractory disease unresponsive to conventional treatment (especially in resource-poor countries like India where biological use is still a daydream for the general population).
